# The association between respiratory tract infection incidence and localised meningitis epidemics: an analysis of high-resolution surveillance data from Burkina Faso

**DOI:** 10.1038/s41598-017-11889-4

**Published:** 2017-09-14

**Authors:** Judith E. Mueller, Maxime Woringer, Souleymane Porgho, Yoann Madec, Haoua Tall, Nadège Martiny, Brice W. Bicaba

**Affiliations:** 1 EHESP French School of Public Health, Sorbonne Paris Cité, Paris, France; 2 Institut Pasteur, Paris, France; 3 École Normale Supérieure, Paris, France; 4 Direction de la lutte contre la maladie, Ministry of Health, Ouagadougou, Burkina Faso; 5 Agence de Médecine Préventive, Ouagadougou, Burkina Faso; 6 UMR6282 BIOGEOSCIENCES, University of Burgundy, Dijon, France

## Abstract

Meningococcal meningitis epidemics in the African meningitis belt consist of localised meningitis epidemics (LME) that reach attack proportions of 1% within a few weeks. A meningococcal serogroup A conjugate vaccine was introduced in meningitis belt countries from 2010 on, but LME due to other serogroups continue to occur. The mechanisms underlying LME are poorly understood, but an association with respiratory pathogens has been hypothesised. We analysed national routine surveillance data in high spatial resolution (health centre level) from 13 districts in Burkina Faso, 2004–2014. We defined LME as a weekly incidence rate of suspected meningitis ≥75 per 100,000 during ≥2 weeks; and high incidence episodes of respiratory tract infections (RTI) as the 5^th^ quintile of monthly incidences. We included 10,334 health centre month observations during the meningitis season (January-May), including 85 with LME, and 1891 (1820) high-incidence episodes of upper (lower) RTI. In mixed effects logistic regression accounting for spatial structure, and controlling for dust conditions, relative air humidity and month, the occurrence of LME was strongly associated with high incidence episodes of upper (odds ratio 23.9, 95%-confidence interval 3.1–185.3), but not lower RTI. In the African meningitis belt, meningitis epidemics may be triggered by outbreaks of upper RTI.

## Introduction

The meningitis belt in sub-Saharan Africa has the highest incidence rate of meningococcal meningitis worldwide. This acute infection of the meninges occurs here with pronounced seasonality (seasonal hyperendemicity), sporadic localised epidemics and epidemic waves which emerge every 7–10 years^1^. The meningitis belt is characterized by six months of dry season and low annual precipitation amounts of 300–1100 mm^2^ during one single rainy season. During a localised meningitis epidemic (LME), the attack proportion can reach around 1% within a few weeks These LME usually do not concern all health centres of a district, but only a small subset^3, 4^. During epidemic waves, the frequency of LME is increased during 1–3 consecutive years. In any instance, case fatality is about 10% despite timely treatment and 20% of survivors have long-term psychomotor sequelae^5^.

The mechanisms behind the meningitis belt epidemiology are only partially understood. While the seasonal hyperendemicity is most likely related to the climatic conditions (low relative air humidity and dust-loaded wind)^6, 7^, the occurrence of epidemic waves could in part be explained by meningococcal strain evolutions and population immunity^8–10^. However, the factors leading to LME remain unknown. A hypothetical explanatory model proposed that a viral respiratory infection epidemic in a given population may act as an epidemic co-factor^1^, by rapidly increasing meningococcal transmission and acquisition in the nasopharynx. This would lead to a proportional increase in disease, on the background of high risk of invasive disease given carriage during the dry season^11^. If this hypothesis was correct, routine surveillance data of meningitis-belt countries would show an association between episodes of high incidence rates of acute respiratory infection and the occurrence of localised meningitis epidemics during the dry season. To explore this hypothesis, we conducted an ecological study based on routinely notified respiratory infections and bacterial meningitis from Burkina Faso.

Burkina Faso, which lies in the meningitis belt, introduced a meningococcal serogroup A conjugate vaccine by mass campaign into the 1- to 29-year-old population during 2010^12^. This vaccine introduction appears to have eliminated serogroup A meningitis epidemics, while epidemics due to other serogroups (W, X, C) continue to occur^9, 13^. Burkina Faso also introduced *Haemophilus influenzae* type b and pneumococcal conjugate vaccine into the Expanded Program on Immunization in 2006 and 2013 respectively. These pathogens are, not involved in meningitis epidemics, although pneumococci substantially contribute to the seasonality of bacterial meningitis and cause outbreaks. Meningitis epidemics remain a major burden to the population and understanding the pathophysiological mechanism behind the phenomenon is needed to design appropriate and sustainable prevention strategies.

## Methods

### Data compilation

**Figure 1:**
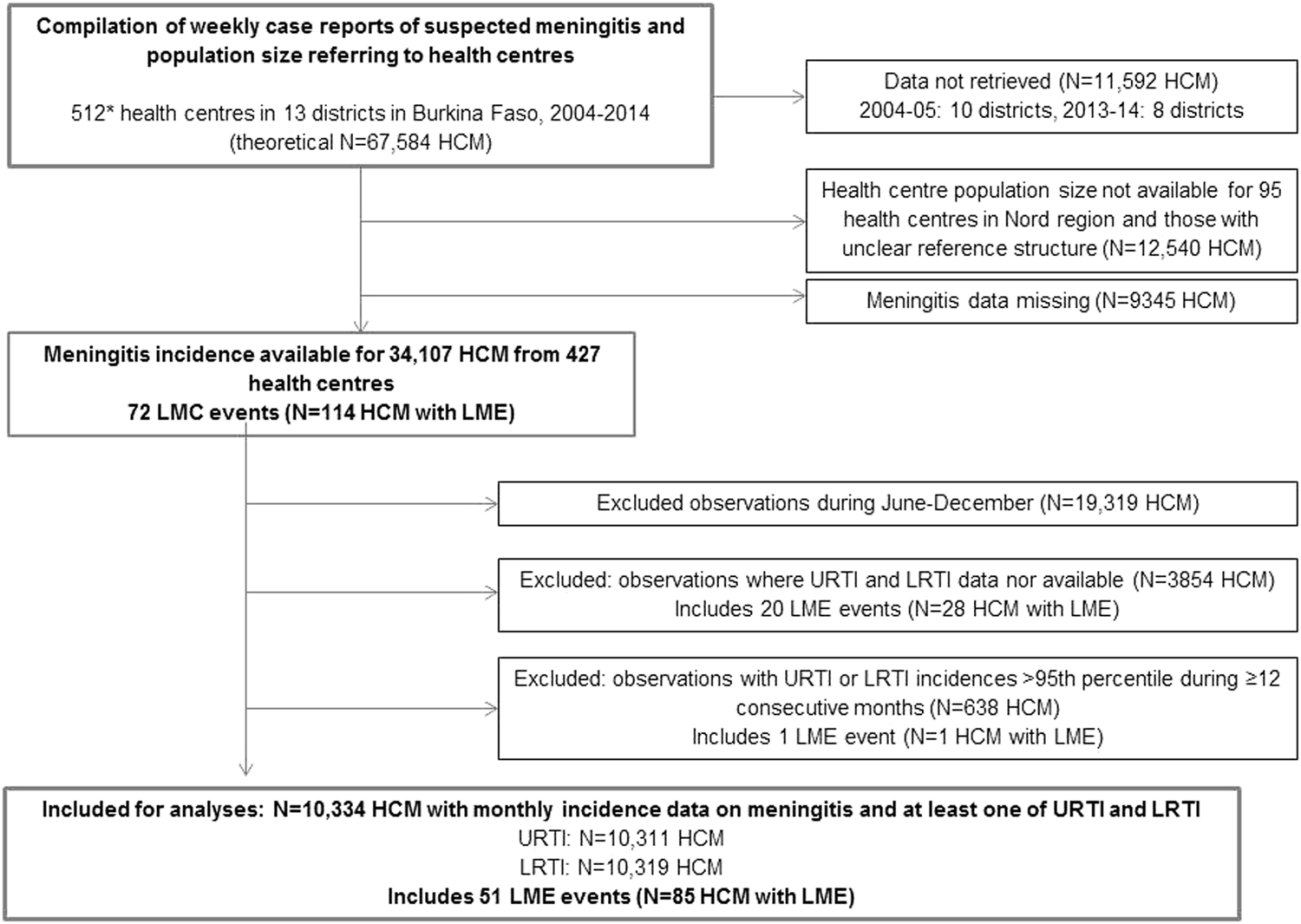
Flow chart of data compilation, exclusion and inclusion. *number of health centres increasing over years, thus a substantial amount of HCM is registered as missing. HCM, health centre months. LME, localised meningitis epidemic. URTI/LRTI, upper/lower respiratory tract infections.

We used routine surveillance data at health centre resolution from 13 health districts in Burkina Faso, including weekly case counts of suspected acute bacterial meningitis^3^ and monthly case counts of clinically suspected upper (URTI) and lower (LRTI) respiratory tract infections. According to the national surveillance guidelines, URTI cases included otitis, severe sore throat and rhinopharyngitis; LRTI cases included pneumonia, bronchitis and bronchopneumonia. Two health regions (Hauts-Bassins and Nord) and two additional health districts (Boulsa in Centre-Nord region and Dédougou in Boucle du Mouhoun region), were selected for pre-existing research collaboration and history of occurrence of epidemic events. We contacted their district statistical offices to retrieve health centre level data for the period 2004 through 2014. For one of five districts in the Nord region, no data on respiratory infections could be retrieved for any year (Yako). Overall, 13 districts provided thus data (Fig. 1). Due to logistical constraints, 2004–2005 data were collected for three districts (Houndé, Orodara, Séguenéga) only, and 2013–2014 data for five districts (Boulsa, Dédougou, Houndé, Orodara, Séguenega), only. These districts are situated across the four regions. Ninety-five health centres in the Nord region were excluded as population size was unknown. Another 13 district years were excluded due to missing data on respiratory infections. The data used in this project had previously been collected by health authorities for the purpose of routine surveillance and disease control. The data consists of aggregated case counts without any individual-level information. As standard clinical procedures were not changed by routine surveillance and individual data were not recorded, no informed consent had been collected from patients. The present project of data analysis received authorisation from the Department of Disease Control (Direction de la lutte contre la maladie, DLM) at the Ministry of Health, Burkina Faso.

To validate the resulting database, we aggregated the observations into district weeks and compared them to the number of weekly cases per district routinely reported by the Ministry of Health to the World Health Organization. Based on over 4,000 district weeks, 62% of the health centre weeks showed a mismatch of ≤2 cases and 93% of ≤5 cases (median error: 0).

### Definition of outcome and exposure variables

The incidence threshold recommended by WHO for epidemic response (10 per 100,000) is applied at the district level. By contrast, to identify individual localised epidemic events, we defined LME based on a threshold of weekly incidence rate at the health centre level, as previously reported^4^. The best performance in identifying epidemic annual incidences of ≥0.8% in individual health centre was obtained with a weekly threshold of ≥75 cases per 100,000 inhabitants during at least two consecutive weeks with at least 5 cases per week (sensitivity 98.5%, specificity 98.0%). Because URTI and LRTI data were available at monthly intervals only, months were categorized according to the identification of at least one week of LME. For a sensitivity analysis, we defined LME as a monthly meningitis incidence of 125 cases per 100,000. We assigned a predominant serogroup to each LME based on district level laboratory reports by the Ministry of Health. Because LE were identified only during January-May, we restricted the analyses to this period.

To avoid bias due to certain surveillance practices or population size estimation error, we excluded 722 health centre months which reported incidences of URTI or LRTI above the 95^th^ percentile for >12 consecutive months. We categorised URTI and LRTI monthly incidences by quintiles across all health centre months, and considered episodes of URTI and LRTI above the 5^th^ quintile (≥2.47 per 1000 inhabitants for URTI and ≥13.47 for LRTI) as high incidence episodes. Primary analyses took into account URTI, LRTI and LE observations during the same month. We created a secondary exposure variable to evaluate the association between LE during a given month and the level of URTI and LRTI during the month prior. We further differentiated months with first occurrence of LRTI or URTI incidences in the 5^th^ quintile, from those where high incidences were already observed during the previous month. Finally, we conducted separate analyses grouping years with high serogroup A incidence (206–2008) and those with predominance of serogroups W and X (2010–2014). For these additional analyses, lower quintiles were collapsed to obtain sufficient statistical power.

### Statistical analyses

As the outcome was epidemic/not epidemic and the data contained a spatial structure with monthly observations clustered within health centres, which lie within districts, we used a mixed-effects model for binary distribution specifying nested random effects for health centre and for districts with unstructured covariance (Stata command *melogit*). In the main analysis, a fixed effect for calendar year was introduced. In an alternative analysis, we included a crossed effects component with calendar year as a third random effect; this model required substantially greater computational effort, but yielded similar odds ratios (<10% difference). All analyses were conducted in Stata/IC14.1 (StataCorp. 2015).

### Confounding variables

Potential confounders of the association between URTI or LRTI episodes and LME could be factors that increase transmission or disease susceptibility for meningococcal and respiratory disease pathogens locally and for a limited duration. We therefore included dust conditions, relative air humidity and calendar month into the multivariate models. For dust conditions, we used the MODIS deep-blue aerosol optical thickness (AOT) product at a daily time-step, which has been shown to be a good proxy for the dust load on the ground surface in Burkina Faso during the dry season^14^. The deep blue AOT, initially available at a 10 km-spatial resolution, were subsampled on a 1 km × 1 km grid, and pixels lying within the health centre coverage region were averaged. Values were further averaged on a monthly scale. We categorised monthly AOT values in quartiles across health centre months from January through to May. Data on monthly means of relative air humidity for the main city of each health region were extracted from the website tutiempo.net (http://tutiempo.net). No data were available for the Centre-Nord region (Boulsa district). We used three categories of relative air humidity <20%, 20–39.9% and ≥40%.

### Data availability statement

The data that support the findings of this study are available from Direction de lutte contre la maladie (DLM), Ministry of Health of Burkina Faso, but restrictions apply to the availability of these data, which were used under license for the current study, and so are not publicly available. Data related to the present work are however available from the authors upon reasonable request and with permission of DLM, Ministry of Health of Burkina Faso. For this purpose, please contact the corresponding author JEM.

## Results

**Figure 2:**
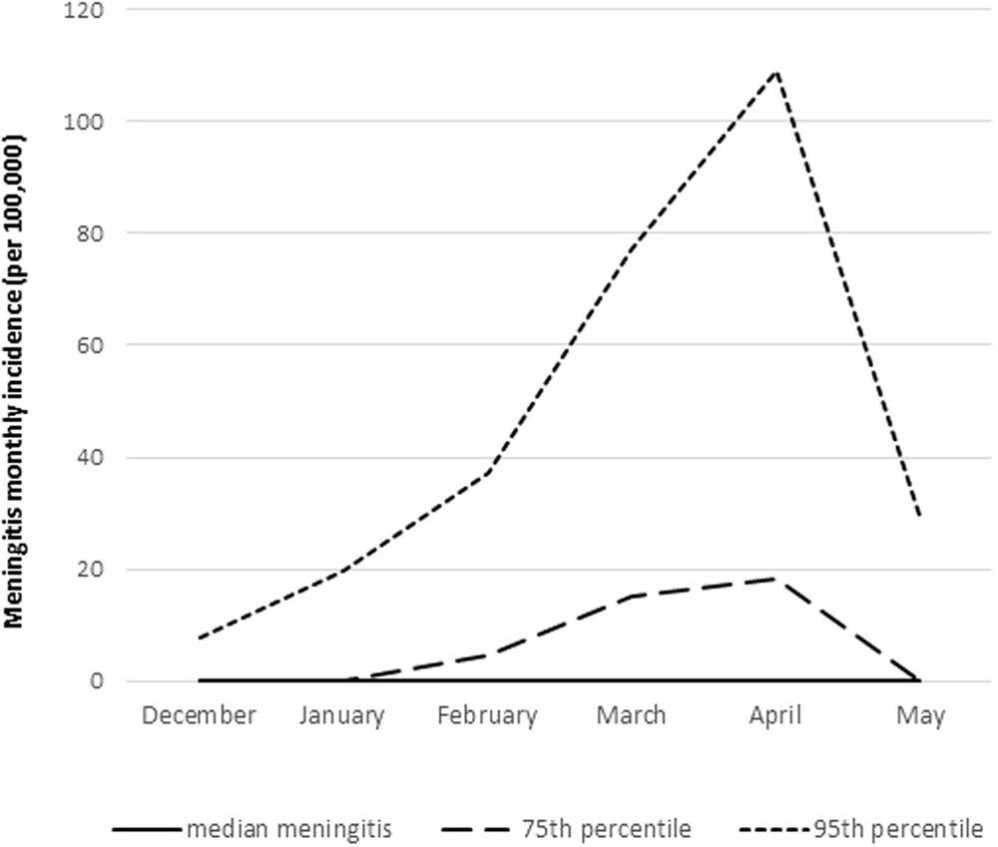
Monthly incidence of suspected meningitis at the health center level during the meningitis season (December–May) in 13 districts of Burkina Faso, 2004–2014. The lines represent median, 75th and 95th percentile. Median incidences are 0 and thus not visible.

Among the 512 health centres included, which corresponded to 67,584 health centre months theoretically targeted, meningitis or population size data were not be retrieved for 33,477 health centre months (Fig. 1). Reasons included partial collection for most ancient and most recent years, missing population size (in particular for private clinics or regional reference hospitals) and missing meningitis data (in particular for health centre months prior to the creation of a new health centre). Meningitis incidence data were thus available from 427 health centres (34,107 health centre months), during which 15,420 suspected meningitis cases were reported. The typical incidence peak at the end of March was found in all districts, but declined over the observed time period (Fig. 2, and supplementary material S1 and S2a **)**. Particularly high incidences were observed during the epidemic wave from 2006 to 2008. We identified 72 LME, corresponding to 114 health centre months. Until 2008, all identified LME were related to serogroup A. After 2011, following serogroup A conjugate vaccine introduction, only serogroups W and X were found.

**Figure 3:**
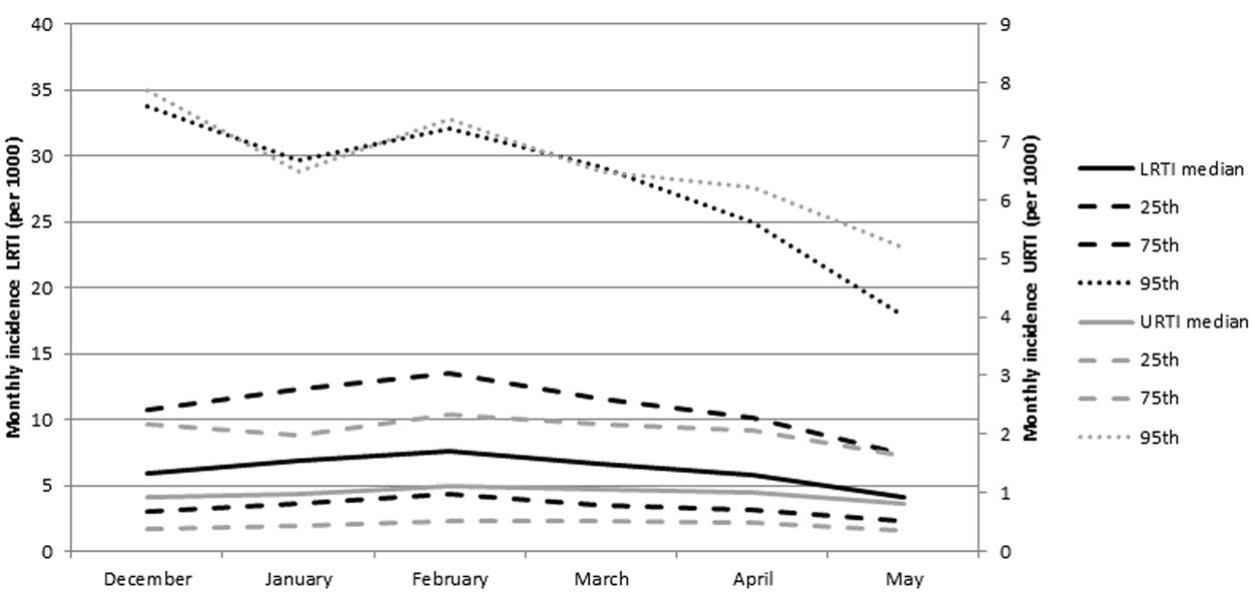
Monthly incidence of upper (URI) and lower (LRI) respiratory infections during December–May in 13 districts of Burkina Faso, 2004–2014. The lines represent median, 25th and 75th percentile.

**Figure 4:**
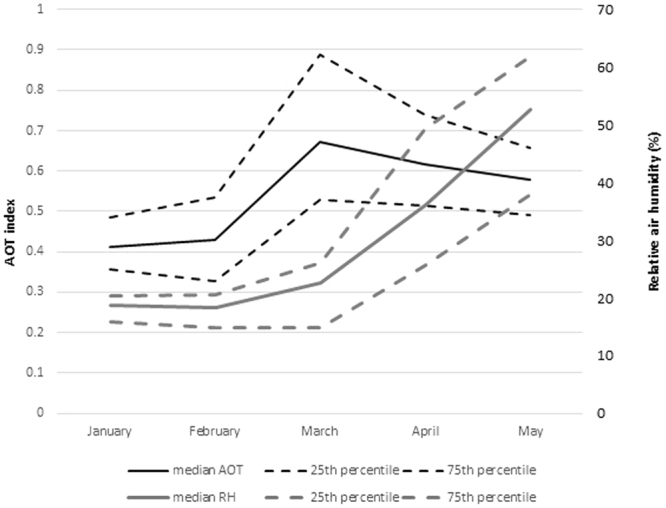
Average monthly aerosol optical index (AOT) and relative air humidity (RH) during January–May in 13 (model including RH: 12) districts of Burkina Faso, 2004–2014. The lines represent median, 25th and 75th percentile.

Overall, 401,110 URTI cases and 1,681,212 LRTI cases were reported during the observation period, with monthly incidence rates showing two seasonal peaks in October and February (supplementary material Figure S2b,c). Half (44% and 46%, respectively) of URTI and LRTI cases were reported from January through to May, even though incidences did not vary substantially across months during this period (Fig. 3). About half of the high URTI incidence episodes lasted for four months or longer. AOT showed the typical maximum during the month of March^15^ and relative air humidity increased from January towards the end of the dry season in May (Fig. 4).

For the period between January and May, we excluded health centre months with continuous peak URTI or LRTI incidences. We retained 10,334 health centre months for which data on meningitis and URTI or LRTI incidence were available (Fig. 1). These included 10,311 health centre months for URTI and 10,319 health centre months for LRTI; and 51 LME events, corresponding to 85 health centre months with LME (Table 1).

**Table 1:** Characteristics of localised meningitis epidemics (LME) identified in 13 health districts, Burkina Faso, 2004–2014. LE were defined as ≥75 cases per 100,000 inhabitants during at least two out of four consecutive weeks and with ≥5 cases per week. Table shows only LME where data on respiratory infection incidence was available.

**Year**	**District**	**Number of HC with LME episodes**	**Number of weeks during which the LME definition was met in the HC**	Predominant meningococcal serogroup during LME*
			median (range)	
2005	Orodara	1	1 (1)	ND
2006	Dafra	1	4	A
	Dandé	2	2 (1–3)	A
	Houndé	8	3 (1–5)	A
	Karangasso	3	8 (3–12)	A
	Lena	6	5 (2–8)	A
	Orodara	1	2	A
	Seguenega	6	2.5 (1–4)	A
	Titao	3	3 (1–6)	A
2007	Boulsa	2	5 (2–8)	A
	Dandé	1	4	A
	Houndé	1	2	A
	Orodara	3	4.5 (2–6)	A
	Seguenega	2	2.5 (2–3)	A
	Titao	2	2.5 (2–3)	A
2008	Boulsa	2	4.5 (4–5)	A
	Orodara	2	3.5 (2–5)	A
	Seguenega	1	1	A
	Seguenega	2	1.5 (1–2)	X
	Titao	1	3	A
2014	Houndé	1	3	W

HC, health centres. ND, not defined. *according Ministry of Health, Epidemiological surveillance department Burkina Faso.

An LME was present during 0.8% of the health centre months considered, and this proportion increased from 0.2 to 1.8% across URTI quintiles (Table 2). The corresponding crude odds ratios (95% confidence interval (CI)) showed an increased risk of LME with increasing URTI incidence quintiles, with an OR of 10.10 (3.25–31.34) in the 5^th^ quintile (Table 3) and a significant effect from the 3^rd^ quintile onwards. After multiple adjustments, odds ratios were higher, however with wider confidence intervals. The crude odds ratio (95% CI) tended to be substantially but not significantly greater when a high incidence episode had been observed the month prior (12.23 (3.86–38.75)) compared to first-time episodes (6.07 (1.61–22.86)) (supplementary material Table S1). A weaker association was observed with a one month lag, linking LME to high URTI incidence episodes during the previous month (2.44, 1.07–5.59) (supplementary material Table S1). Strong associations were observed in analyses including the period between 2006 and 2008, where epidemic waves were due to serogroup A (5^th^ vs 1^st^−4^th^ quintile, crude OR 3.26, 95% confidence interval 1.62–6.57) or for the period from 2010 onwards, when only serogroups W and X were found (14.22, 0.91–223.18) (supplementary material Table S1). In the sensitivity analysis using a less specific but more sensitive LME definition of 125 monthly cases per 100,000, a similar increase of odds ratios across URTI quintiles was observed, with an OR of 4.89 (2.55, 9.38) for the fifth quintile.

**Table 2:** Health centre months with and without localised meningitis epidemics (LME) by different categories of monthly incidence of upper and lower respiratory tract infection (URTI, LRTI). Burkina Faso, 2004–2014.

	**URTI**			**LRTI**		
**Quintiles of incidence rate (per 1000)**		No LME month (N = 10,226)	LME month (N = 85)		No LME month (N = 10,234)	LME month (N = 85)
1^st^ quintile	<0.39	2213 (98.8)	4 (0.2)	<2.98	2382 (99.2)	20 (0.8)
2^nd^ quintile	0.39-<0.78	2079 (99.6)	8 (0.4)	2.98-<5.37	2171 (99.4)	14 (0.6)
3^rd^ quintile	0.78-<1.33	2047 (99.9)	22 (1.1)	5.37-<8.22	1999 (98.7)	6 (0.3)
4^th^ quintile	1.33-<2.47	1996 (99.2)	16 (0.8)	8.28-<13.47	1862 (98.6)	26 (1.4)
5^th^ quintile	≥2.47	1891(98.2)	35 (1.8)	≥13.47	1820 (99.0)	19 (1.0)

N (%).

**Table 3:** Association between high incidence episodes of upper respiratory tract infections (URTI) and occurrence of localised meningitis epidemic, Burkina Faso, 2004–2014. All models are mixed-effect logistic regression accounting for spatial data structure. The model adjusting for relative air humidity (RH) includes only 12 districts.

	Crude	Adjusted for AOT, RH and month	Adjusted for AOT, RH, month and year
**URTI monthly incidence rate (per 1000)**			
<0.39	1	1	1
0.39–<0.78	2.25 (0.66–7.65)	5.21 (0.61–44.04)	3.68 (0.39–34.69)
0.78–<1.33	6.85 (2.26–20.72)	15.58 (2.01–120.67)	20.11 (2.28–177.58)
1.33–<2.47	5.15 (1.62–16.30)	8.18 (1.02–65.57)	16.10 (1.73–149.83)
≥2.47	10.10 (3.25–31.34)	23.94 (3.09–185.30)	54.55 (6.00–495.63)
Monthly mean AOT (quartiles)			
<0.40		1	1
0.40–<0.51		0.89 (0.33–2.36)	4.36 (1.04–18.25)
0.51–<0.66		1.57 (0.62–3.98)	2.11 (0.42–10.69)
≥0.66		1.64 (0.63–4.29)	6.10 (1.00–37.09)
Monthly mean RH			
≥40%		1	1
20–39.9%		0.68 (0.26–1.74)	1.23 (0.38–4.00)
<20%		2.13 (0.61–7.47)	0.51 (0.12–2.21)
Month			
January		1	1
February		3.88 (1.04–14.54)	3.06 (0.71–13.14)
March		11.58 (2.99–44.83)	4.62 (0.75–28.69)
April		15.65 (3.23–75.78)	3.17 (0.39–25.79)
May		1.80 (0.24–13.35)	0.22 (0.02–2.33)
Year *			
2005			1.97 (0.09–43.39)
2006			171.98 (16.52–1790.36)
2007			17.76 (1.56–201.83)
2008			2.94 (0.25–35.06)
2010			2.49 (0.20–30.48)
2014			1

Odds ratio and 95% confidence intervals AOT, aerosol optical thickness. *Years 2004, 2009, 2011–13 predicts failure perfectly, dropped from model.

An LME was present in 0.3 to 1.4% of health centre months across quintiles of LRTI (Table 2), however without any trend. The odds ratios (95% CI) varied from 0.41 (0.16–1.06) to 1.89 (0.97–3.69), without any trend (Table 4). A strong and significant association was observed from the fourth LRTI incidence quintile upwards only when adjusting for calendar year (5^th^ quintile, 6.68 (1.94–23.07)) (Table 4). However, in analyses separating periods 2006–2008 and 2010–2014, the 5^th^ quintile was significantly associated with LME occurrence, compared to other quintiles (supplementary material Table S1). The association between LME and the 5^th^ LRTI quintile was weaker when the high incidence episode existed already during the month prior compared to first-time events (supplementary material Table S1).

**Table 4:** Association between high incidence episodes of lower respiratory tract infections (LRTI) and occurrence of localised meningitis epidemics, Burkina Faso, 2004–2014. All models are mixed-effect logistic regression accounting for spatial data structure. The model adjusting for relative air humidity (RH) includes only 12 districts.

	Crude	Adjusted for AOT, RH, and month	Adjusted for AOT, RH, month and year
**LRTI monthly incidence rate (per 1000)**			
<2.98	1	1	1
2.98–<5.37	0.93 (0.45–1.94)	0.78 (0.35–1.77)	1.76 (0.61–5.07)
5.37–<8.22	0.41 (0.16–1.06)	0.25 (0.08–0.77)	0.66 (0.17–2.66)
8.28–<13.47	1.89 (0.97–3.69)	1.05 (0.48–2.28)	4.00 (1.35–11.89)
≥13.47	1.05 (0.59–2.21)	0.53 (0.22–1.26)	6.68 (1.94–23.07)
Monthly mean AOT			
<0.40		1	1
0.40–<0.51		0.87 (0.33–2.32)	4.37 (1.06–18.09)
0.51–<0.66		1.42 (0.56–3.58)	1.93 (0.39–9.56)
≥0.66		1.68 (0.65–4.39)	5.85 (0.99–34.40)
Monthly mean of RH			
≥40%		1	1
20–<40%		0.61 (0.24–1.54)	0.93 (0.28–3.05)
<20%		1.94 (0.58–6.53)	0.52 (0.12–2.30)
Month			
January		1	1
February		4.16 (1.11–15.53)	3.01 (0.71–12.65)
March		11.37 (2.94–43.89)	5.13 (0.84–31.18)
April		14.23 (3.01–67.16)	4.18 (0.53–33.12)
May		1.43 (0.20–10.44)	0.29 (0.03–3.00)
Year*			
2005			2.10 (0.10–45.98)
2006			173.33 (16.18–1856.81)
2007			17.53 (1.50–204.51)
2008			3.34 (0.28–40.48)
2010			1.88 (0.15–22.91)
2014			1

Odds ratio and 95% confidence intervals AOT, aerosol optical thickness. *Years 2004, 2009, 2011–13 predicts failure perfectly, dropped from model.

## Discussion

In this analysis of health centre surveillance data from Burkina Faso spanning a decade, we found that high incidence episodes of URTI were strongly associated with the occurrence of localised meningitis epidemics. The association with LRTI was inconsistent and only seen after adjusting for calendar year.

These findings strengthen the hypothesis that acute respiratory tract infections act as co-factors for the occurrence of localised meningitis epidemics in the meningitis belt^1, 16^. The trend of a gradually increasing risk of LME with increasing URTI incidence or duration, the persistence of the association with a time lag and the strength of the observed association may be considered as arguments for a causal relation.

At the individual level, (viral) inflammation of the nasopharygeal mucosa facilitates bacterial adhesion and colonization, including that of meningococci^17, 18^, which can result in increased transmission in the community. This effect is supported by associations between meningococcal carriage and clinical pharyngeal inflammation that had previously been described during meningitis epidemics^19, 20^, in contrast to absence of this association outside epidemics, as reported by a large multi-country carriage study^21^. The biological effect could be emphasized by behavioural changes induced by respiratory infections, such as sneezing and coughing, which can further facilitate meningococcal transmission. At the population level, such accelerated transmission is supported by the over10 times increase in meningococcal carriage prevalence during epidemic situations, as observed by most studies during meningitis epidemics^11, 20^. According to the hypothetical model cited above^1^, the surge in transmission causes the epidemic increase in meningitis incidence, mediated by a high case-carrier ratio during the dry season^11^. The high risk of invasive disease given carriage, in turn could be related to the damage of the mucosal barrier function during periods of low humidity and high dust load.

Viral especially influenza infections lead to immune depression, which facilitates the development of invasive bacterial disease, including meningococcal disease^22^. Studies during meningitis epidemics in the meningitis belt or outbreaks in Europe have reported strong associations between influenza infection or flu-like symptoms and meningitis^15, 23, 24^. While we believe that accelerated bacterial transmission should be the main mechanism behind the hypothesised link between RTI and meningitis epidemcis, this effect could further accelerate the emergence of an LME. In our analysis, an association between LRTI and LME was seen only after adjustment for calendar year. This adjustment considerably increased odds ratios for both URTI and LRTI, suggesting negative confounding. Given the limited number of LME included overall, caution is required when interpreting the year-adjusted results. The difference in odds ratio between URTI and LRTI may mean that inflammation of the nasopharynx is required to obtain the epidemiogenic effect in question; or that the incriminated pathogen rarely causes LRTI. However, given the limited quality of the available RTI data, we cannot conclude on the absence of an effect from LRTI.

The limited number of LME did not allow serogroup-specific analysis, but a strong association between URTI and LME was seen during epidemic waves due to serogroup A and epidemics due to serogroups W or X. The recent epidemic wave due to a new meningococcal serogroup C strain in Nigeria and Niger^9^ was constituted of localised epidemics that were spread across the epidemic regions without any systematic pattern^25^. In the light of the present findings, the epidemic emergence could be interpreted as a coincidence between the introduction of a new meningococcal strain and the occurrence of respiratory pathogen outbreaks.

URTI usually show mild clinical symptoms and do not lead to medical consultation, especially in the low resource settings of Burkina Faso. In consequence, our URTI incidences based on consultations are probably largely underestimated. Furthermore, URTI and LRTI reporting may have been neglected during LME due to the strain on the health care system. Both would have led to an underestimated association between URTI or LRTI and LME.

No laboratory information was available for any of the reported respiratory infections. One case-control study during a meningitis epidemic in Chad reported higher prevalence of mycoplasmal and viral infection among cases compared to controls^16^. Investigating the nature of the respiratory pathogen (a specific pathogen or a generic effect of various pathogens) will require clinical studies during LME. In theory, meningococcal colonisation itself could cause URTI symptoms; however, if so, they would be mild and not motivate seeking medical care.

Our study has several limitations. First, we may not have adjusted for all relevant confounding factors. Although the ecological level is appropriate in analysing risk factors for epidemic occurrence, no conclusion on a causal link can be made from this type of study, as several behavioural and biological aspects, which are only available from individual level studies (eg, crowded living conditions, smoking, complement deficiencies), may interplay^26^. Although we had collected information on social events, cultural context and vaccination campaigns with polysaccharide meningococcal vaccines, the exhaustiveness of the data did not allow inclusion in the present analysis.

The routine nature of the data limits the validity of our results. Misclassification between URTI and LRTI is likely, as clinical symptoms overlap. The reporting practice may have evolved over the observed period, in particular for LRTI, targeted for pandemic flu preparedness during that decade. Also, reporting practice probably varied between health regions and districts. Although these effects should have been taken into account by the mixed effects logistic regression models, reporting artefacts may occur. Finally, our analysis assumes that changes in RTI reporting incidence are parallel to changes in the true RTI incidence in the general population, which may not be the case, given social and behavioural variations or seasonality of other diseases.

We have selected aerosol load and relative air humidity to represent climatic influences that could explain the observed association, and we acknowledge that other factors, such as temperature or wind speed could be considered in future analyses investigating the hypothesis.

Our study shows high incidence rates of acute respiratory infections in Burkina Faso, with seasonal peaks. A study in Benin found that an increase of consultations for LRTI occurs during the cold dry season^27^. Further evaluation of the specific burden of respiratory diseases in the meningitis belt and their association with climatic factors is needed.

In conclusion, this study adds evidence to the hypothesis that URTI outbreaks during the dry season function as co-factors for localised meningitis epidemics. Depending on the aetiology, this may open opportunities for the development of serogroup-unspecific preventive interventions at the population level, including vaccination against the involved pathogen. Given the continuing difficulties in preventing and controlling epidemics of all meningococcal serogroups, any additional preventive intervention may be beneficial. Beyond this, the described association represent a striking example of the interactions between climate, bacteria and viruses that threaten human health.

## Electronic supplementary material

Supplementary material
