# Supplementary material for: The association between respiratory tract infection incidence and localised meningitis epidemics: an analysis of high-resolution surveillance data from Burkina Faso
Source: Sci Rep. 2017 Sep 14;7:11570. doi: 10.1038/s41598-017-11889-4 (PMC5599514; doi:10.1038/s41598-017-11889-4)
Supplement: Supplementary file 1 — Supplementary material [file 41598_2017_11889_MOESM1_ESM.pdf]

Supplementary material to

The association between respiratory tract infection incidence and localised meningitis epidemics: an analysis of high-resolution surveillance data from Burkina Faso

Judith E. Mueller[1,2], Maxime Woringer[3], Souleymane Porgho[4], Yoann Madec[2], Haoua Tall[5], Nadège Martiny[6], Brice W. Bicaba[4]

1) EHESP French School of Public Health, Sorbonne Paris Cité, Paris, France
2) Institut Pasteur, Paris, France
3) École normale Supérieure, Paris, France
4) Direction de la lutte contre la maladie, Ministry of Health, Burkina Faso
5) Agence de Médecine Préventive, Ouagadougou, Burkina Faso
6) UMR6282 BIOGEOSCIENCES, University of Burgundy, Dijon, France

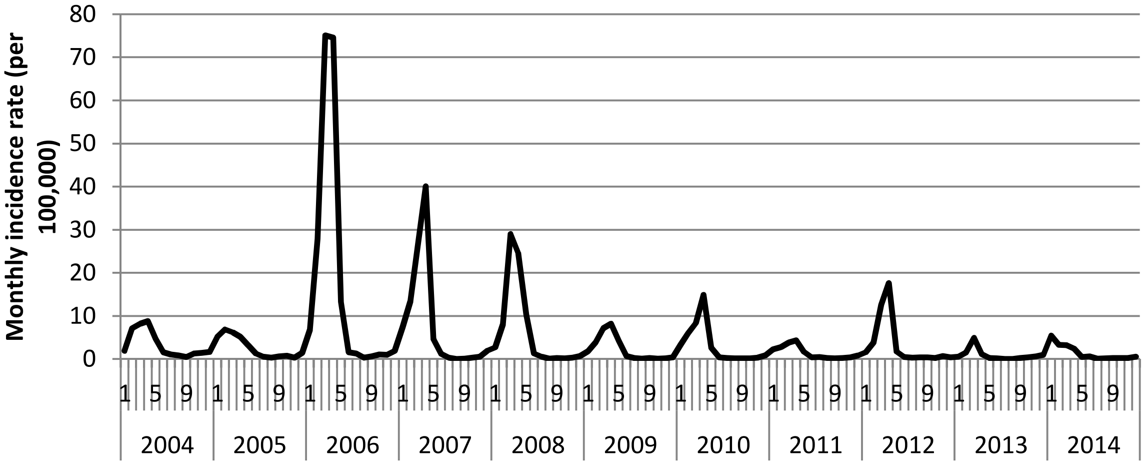

Supplementary Figure S1. Monthly meningitis incidence rates at the district level, 13 district in Burkina Faso, 2004-2014.

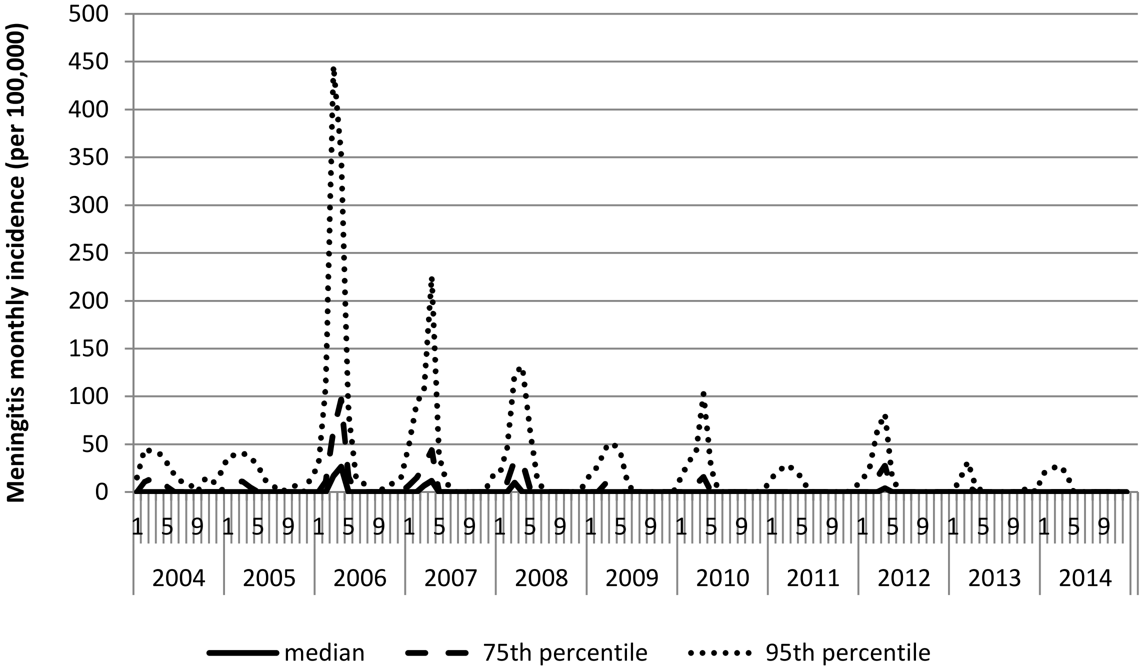
Meningitis monthly incidence (per 100,000)
500
450
400
350
300
250
200
150
100
50
0
2004
2005
2006
2007
2008
2009
2010
2011
2012
2013
2014
median
75th percentile
95th percentile

a.

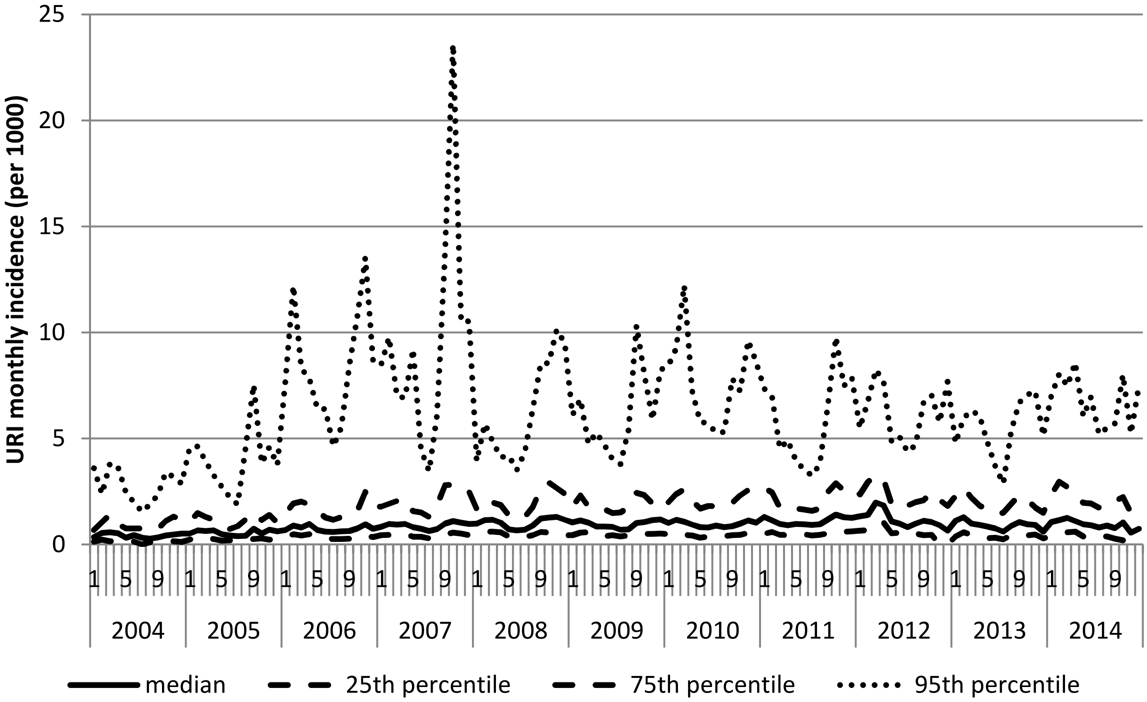
URI monthly incidence (per 1000)
25
20
15
10
5
0
2004
2005
2006
2007
2008
2009
2010
2011
2012
2013
2014
median
25th percentile
75th percentile
95th percentile

b.

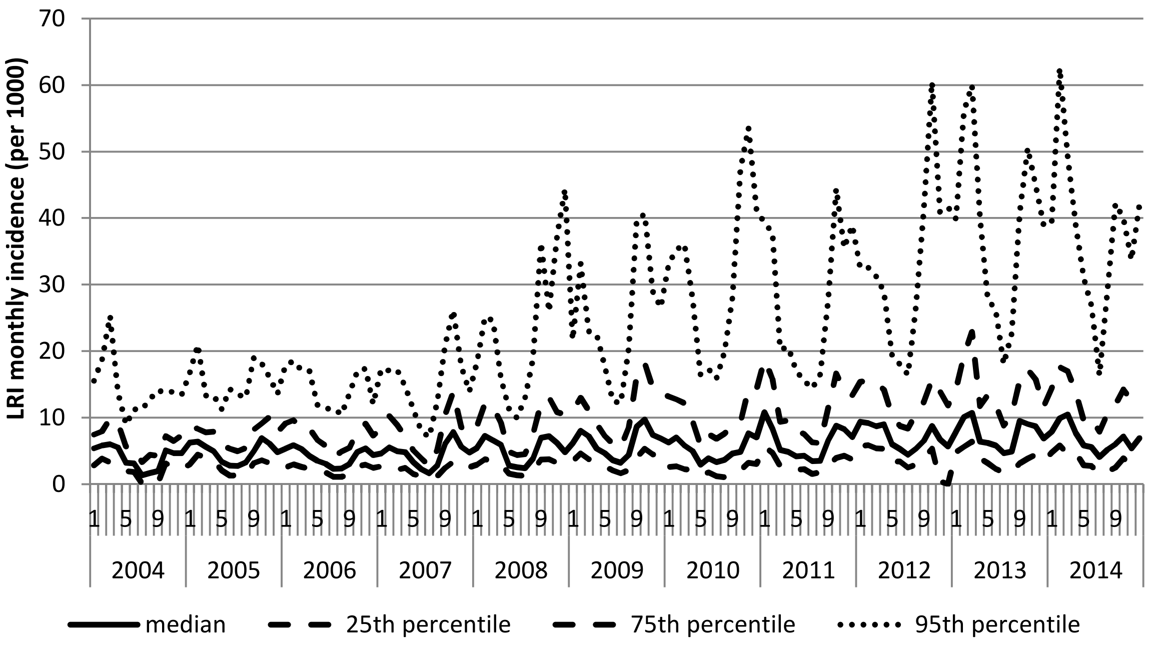

c.

Supplementary Figures S2 a,b and c. Monthly incidence rates of notified suspected acute bacterial meningitis, and upper (URI) and lower (LRI) respiratory tract infections at the health centre level in 13 districts, Burkina Faso, 2004-2014.

Table S1. Association between high incidence episodes of upper (URTI) and lower (LRTI) respiratory tract infections and occurrence of localised meningitis epidemics, Burkina Faso, 2004-2014. All models are crude mixed-effect logistic regression accounting for spatial data structure.

	URTI	**LRTI**
**With one-month lag**		
1st quintile	1	1
2nd quintile	1.63 (0.45-2.66)	2.71 (0.46-15.99)
3rd quintile	1.43 (0.61-3.38)	5.55 (1.03-29.85)
4th quintile	1.43 (0.61-3.38)	2.31 (0.44-12.26)
5th quintile	2.44 (1.07-5.59)	1.56 (0.36-6.74)
**Duration of high incidence episode**		
1st quintile	1	1
2nd to 4th quintile	4.45 (1.56-12.74)	1.04 (0.58-1.85)
5th quintile first time	6.07 (1.61-22.86)	1.84 (0.72-4.71)
5th quintile already during month prior	12.23 (3.86-38.75)	0.73 (0.32-1.68)
**Period 2006 - 2008**		
1st to 4th quintile	1	1
5th quintile	3.26 (1.62-6.57)	2.57 (1.28-5.19)
**Period 2010 - 2014**		
1st to 4th quintile	1	1
5th quintile	14.22 (0.91-223.18)	13.02 (1.20-141.71)
